# Advancement of Computational Design Drug Delivery System in COVID-19: Current Updates and Future Crosstalk- A Critical Update

**DOI:** 10.2174/1871526523666230816151614

**Published:** 2023-08-25

**Authors:** Abu Mohiuddin, Sumanta Mondal

**Affiliations:** 1 Department of Pharmaceutical Science, GITAM School of Pharmacy, GITAM (Deemed to be University), Visakhapatnam-530045, A.P., India

**Keywords:** COVID-19, computational drug design, SARS-CoV-2, artificial intelligence structure-based, delta divergent, utilized computational or computer-aided approaches

## Abstract

Positive strides have been achieved in developing vaccines to combat the coronavirus-2019 infection (COVID-19) pandemic. Still, the outline of variations, particularly the most current delta divergent, has posed significant health encounters for people. Therefore, developing strong treatment strategies, such as an anti-COVID-19 medicine plan, may help deal with the pandemic more effectively. During the COVID-19 pandemic, some drug design techniques were effectively used to develop and substantiate relevant critical medications. Extensive research, both experimental and computational, has been dedicated to comprehending and characterizing the devastating COVID-19 disease. The urgency of the situation has led to the publication of over 130,000 COVID-19-related research papers in peer-reviewed journals and preprint servers. A significant focus of these efforts has been the identification of novel drug candidates and the repurposing of existing drugs to combat the virus. Many projects have utilized computational or computer-aided approaches to facilitate their studies. In this overview, we will explore the key computational methods and their applications in the discovery of small-molecule therapeutics for COVID-19, as reported in the research literature. We believe that the true effectiveness of computational tools lies in their ability to provide actionable and experimentally testable hypotheses, which in turn facilitate the discovery of new drugs and combinations thereof. Additionally, we recognize that open science and the rapid sharing of research findings are vital in expediting the development of much-needed therapeutics for COVID-19.

## 1 INTRODUCTION

The SARS-CoV-2, which is severe acute respiratory syndrome coronavirus 2 that roots the evolving 2019 coronavirus ailment (COVID-19), which shares structural resemblances with SARS-CoV-1 also positions a thoughtful threat to comprehensive public healthiness. Air droplets, among other things, can disseminate SARS-CoV-2 [1]. The number of cases and fatalities dramatically amplified due to COVID-19's high infection rate. As per the WHO (World Health Organization), there were 37,58,560 fatalities and 174 061 995 confirmed cases as of June 10th, 2021. One of the best ways to restore order to human civilization is through vaccinations. Several vaccinations have received emergency use authorization. Although vaccinations have had very beneficial results in many nations, they continue to confront several important obstacles. A few patients were found to suffer thrombocytopenia and venous thrombosis, subsequently getting the first dosage of the SARS-CoV-2 vaccination for COVID-19, according to studies [2-5]. Additionally, not everyone benefits fully from vaccination protection. For instance, in the third phase, 72.8% and 78.1%, respectively, of the 2 whole-virus incapacitated vaccines invented by the China National Biotech (CNB) Group Company Limited were effective against indicative COVID-19 cases [6, 7]. The vaccines' ability to guard against newly evolving SARS-CoV-2 transformations is the last and most crucial issue. Today, delta has quickly risen to prominence as the predominant SARSCoV-2 type. Research indicates that vaccines provide significantly less protection against delta, and vaccinated individuals can spread the delta form when they develop breakthrough infections [8, 9]. It is unknown whether immunizations can stop the spread of delta. Drug discovery is yet another crucial method of viral defense. Drug repositioning has been a crucial area of study for pharmaceuticals. Recent months have seen the advancement of novel healing targets besides the identification of previously unknown connections between diseases that appeared to be unrelated in the exploration for medications to combat COVID-19. But most medications, including remdesivir, dexamethasone, and hydroxychloroquine, don't work well in treating COVID-19 [10]. According to structural biology and molecular physics, creating unique therapeutic medications is a trend [11, 12]. In complex, rare, chronic, *etc*., diseases, medication design may have a greater beneficial impact than medication passage and amalgamations [13, 14]. Many specific medications are currently being developed using computer-generated transmission methods grounded on particular pharmacologic considerations or an endwise agenda to manage molecule production [15-20]. Frequent new advancements have been executed in designing drugs in the wide-ranging practice of medicinal interaction by altering physical properties [21, 22], refining effectiveness, and eliminating or amending toxicophores [23, 24] as a consequence of the exhaustive study of illnesses and investigation [25] of the construction of aim proteins [26-28]. Every drug design is based on how a medicine interacts with its target (usually proteins). Therefore, developing techniques [29, 30] for estimating the strength of interactions between proteins and ligands can increase the effectiveness of drug development [31-34]. Drug discovery stages involve structural optimization of lead compounds to achieve the peak level of discrimination, influence, and acceptable pharmacokinetic and physicochemical features [26, 35, 36]. Importantly, examining protein surface binding hotspots can direct the investigation of possible ligand-binding sites. The spread of the SARSCoV-2 virus is a catastrophe for the entire planet. When developing medications for new targets, many strategies are constrained by a lack of data [37-42]. The use of machine knowledge and deep education as drug design algorithms has increased over the past few decades as large data and computer technologies have advanced [43-46]. Deep learning has increased its involvement in the creation and design of drugs, including the planning of chemical syntheses [44, 47-54], identification of drug-target interactions [49-51], and prediction of molecular characteristics and activity [47, 48]. Consequently, the latent deep knowledge and molecular modeling methodologies aid in developing drug design pipelines, particularly when target-specific ligand databases are scarce or non-existent [55, 56]. The intended medication must have a potent disease-inhibiting impact. Nevertheless, clinical trial failure can be prevented by predicting the pharmacokinetics and toxicity properties of the planned medicine [57, 58]. To advance structure-based or sequence-based drug design matters, techniques, including silicon and deep learning, are now being applied [19, 59-64]. Consequently, with the development of biometrics and bioinformatics, medication design is being rapidly applied on a larger scale [65-67]. The systematic creation of molecules with biological activity as potential therapeutic candidates is now possible using effective methods [68-70]. Countless epidemic diseases have been countered by strategies based on drug design [38, 71-74]. Let's say a multiplicative adversarial autoencoder [75], which syndicates a neural system with simulated transmission of a biochemical database, was invented to advise forthcoming HIV-1 entrance inhibitors appropriate to avert the early infection of HIV. But because COVID-19 ravages the world, scientists are concentrating on developing drugs to combat SARS-COV-2.

## 2 LITERATURE RESEARCH BACKGROUND INFORMATION

A case study presents a comprehensive evaluation of the dynamic impact of the COVID-19 pandemic on the environment, with a specific focus on China. The analysis investigates the short-term improvements in air quality and the significant contributions to global carbon emission reduction observed during the initial outbreak. Furthermore, it examines the potential implications in the long run, considering the lifting of lockdown measures and the resumption of large-scale industrial production, which may lead to a resurgence in energy use and greenhouse gas (GHG) emissions surpassing pre-pandemic levels. In addition, the COVID-19 pandemic has had a notable effect on the concentration of nitrogen dioxide (NO2) in the atmosphere, particularly in regions such as Wuhan, China. As the pandemic unfolded, economic activities were curtailed, and traffic restrictions were imposed, leading to a decline in energy consumption and subsequent improvements in air quality [76]. This phenomenon highlights how reduced economic activities and transportation restrictions have directly contributed to environmental pollution prevention. It is noteworthy that China has been the first major economy to exhibit signs of recovery following the slowdown induced by the COVID-19 pandemic. This study aims to investigate the implications of China's post-COVID-19 economic recovery on the economic growth and energy consumption of other countries, utilizing global VAR quarterly data. In the long run, the spillover effects of China's economic growth are most noticeable in the economic growth of upper-middle-income countries, with an estimated impact of 0.17%. Lower-middle-income countries follow closely with a growth impact of 0.16%, while high-income countries experience a slightly lower impact of 0.15% [77]. The COVID-19 pandemic is a major global challenge that rivals the impact of the 1918 Flu pandemic. It poses significant obstacles to the achievement of the Sustainable Development Goals (SDGs), requiring a thorough examination of its impact on sustainability. While research in developed countries dominates the field, it is crucial to recognize that developing countries face even greater challenges to their sustainable development due to the pandemic. Developed countries prioritize studying education sustainability, while developing countries tend to focus more on economic sustainability during this crisis. Cluster analysis reveals that the COVID-19 pandemic has had adverse effects on all 17 SDGs goals, yet it also presents opportunities for progress in 14 other SDGs goals [78]. Studies examining the effects of the COVID-19 pandemic on carbon emissions have predominantly relied on analyzing year-to-year changes. However, a novel study introduced an alternative method to assess this impact by comparing carbon emissions during the pandemic to projected emissions in the absence of the pandemic. This approach employed scenario analysis to calculate the variance between actual carbon emissions in 2020 and carbon emissions that would have occurred without the pandemic. The simulation exhibited an average relative error of approximately 1%, indicating reliable results. Notably, the scenario simulation of carbon emission reductions in the United States and the European Union closely aligned with the inter-annual change rates of carbon emissions reported by existing statistical data. The simulations conducted for carbon emission reduction in China and India indicate a 5% difference compared to the reported inter-annual change rate of carbon emissions in existing statistics [79]. The integration of digital technologies and public health, specifically in the field of digital healthcare, has played a crucial role in combating the Coronavirus Disease 2019 (COVID-19) pandemic. This pandemic is considered the most significant public health crisis humanity has faced since the 1918 Influenza Pandemic. To gain a better understanding of digital healthcare, a comprehensive and systematic review was conducted, aiming to provide insights and assistance in our fight against the COVID-19 pandemic. Notably, big data, artificial intelligence, cloud computing, and 5G have emerged as highly effective tools in our arsenal to combat the current health crisis. Various applications have demonstrated the indispensable role of these technologies in controlling the spread of COVID-19 [80]. However, most of the existing research on COVID-19 and pollution has adopted a linear perspective, overlooking the nonlinear relationship between these two factors. In light of this, a recent study was conducted to systematically investigate the nonlinear impact of COVID-19 lockdown on four common pollutants (NO2, PM2.5, O3, and SO2) in eight selected cities: Wuhan (China), New York (United States), Milan (Italy), Madrid (Spain), Bandra (India), London (United Kingdom), Tokyo (Japan), and Mexico City (Mexico). The study utilized updated data and employed the Spearman correlation function model. To a certain extent, the global lockdown imposed due to the COVID-19 pandemic resulted in a reduction of nitrogen dioxide and particles. However, it did not have a significant impact on reducing ozone levels [81]. The COVID-19 epidemic has had a profound effect on the worldwide economy and energy markets. To mitigate the shock, stabilize the financial market, and promote economic recovery, the Federal Reserve (Fed) implemented an unlimited quantitative easing (QE) policy. To assess the policy's impact on the energy market during extreme events, this study examined the prices of WTI crude oil and coal from January 1, 2018, to May 7, 2021. The analysis considered the two years preceding the pandemic and divided the pandemic stage into four smaller stages based on the three peaks of the epidemic in the US. The findings from the calculations using the MF-DCCA (Multifractal Detrended Cross-Correlation Analysis) model indicate that coal and WTI crude oil exhibit an interactive relationship. These two energy sources do not simply experience averaged and superimposed risks but rather demonstrate a transmission and interaction of risks. Additionally, the results obtained from the MF-DFA (Multifractal Detrended Fluctuation Analysis) model calculations reveal that the market efficiency experienced a rapid decline in the first quarter of 2020 due to the disruptions in energy supply and demand caused by the epidemic. However, the market efficiency decoupled from the development of the epidemic in the second half of 2020, suggesting a shift in factors influencing market efficiency during that period [82]. Addressing the challenge of combating COVID-19 has become a paramount concern for global public health authorities. To effectively tackle this emerging global issue, it is imperative to delve into structural molecular biology and gain a comprehensive understanding of the life cycle of n-COVID-19. This knowledge will facilitate the discovery and development of drugs and vaccines. Furthermore, it is crucial to remain vigilant about viral mutations across different hosts to implement preventive measures. The outbreaks of zoonotic viruses have resulted in severe human casualties and disrupted global economies, including manufacturing supply chains and reduced market demand. Thus, it is essential to foster collaboration among various institutions, academia, governments, and pharmaceutical companies to contain the current spread of COVID-19 and prepare for future outbreaks. Additionally, further investigation of existing drugs is necessary to enhance prevention and treatment strategies against this infection [1, 82-84].

## 3 STRATEGIES BASED ON DRUG DESIGN FOR COVID-19

The two main types of computational drug strategy approach cast-off for COVID-19 are (i) structure-based methods also (ii) AI-based methods [85, 86]. Nearly techniques combine AI-based and structure-based techniques [87-89].

## 4 STRUCTURE-BASED APPROACHES

For a reason the development of halogen sets in ligands-aim complexes favorably adds to the firmness of the protein-ligand compound, the insertion of halogen molecules on knockout or main complexes has been exploited to leverage their stereo electronic properties [90, 91]. Numerous derivatives have been particularly created to give drug-target communications over their carbamate fraction, as carbamates have become more widely used in medicinal chemistry [92, 93]. Three FDA-approved medications and almost 50 compounds have undergone clinical trials due to fragment-grounded drug origination, which is a successful way of making low molecular weight protein inhibitors and therapeutic applicants [94]. Combinatory interaction and structure-grounded design, which can take advantage of targets and ligands’ basic structural and physicochemical characteristics, must be integrated to permit the transmission of a virtual reference library in the exploration of dynamic molecules. These methods, in our opinion, have proved useful in advancing antiretroviral drugs. The main goal of SBDD is to convert computer-generated ideas into testable experimental procedures while also ranking existing or newly created compounds for desired biological activity. Recent SBDD research for COVID-19 has started to take advantage of cutting-edge DL and AI approaches in addition to utilizing traditional molecular docking and scoring protocols. Researchers have extensively explored drug repurposing (DR) techniques to discover promising pharmacotherapies for the treatment of COVID-19. While various DR approaches have been utilized for different diseases, structure-based DR (SBDR) methods have emerged as a primary therapeutic option against the COVID-19 pandemic. These methods leverage essential information such as the sequence of the severe acute respiratory syndrome coronavirus 2 genomes. In one study, molecular docking was employed to investigate the targeting behavior of N3 against COVID-19 Mpro. The findings demonstrated that N3 exhibited favorable binding within the substrate-binding pocket of the virus. Furthermore, kinetic analysis was conducted to evaluate the efficacy of N3 against COVID-19. The time-dependent irreversible inhibitory behavior of the N3 compound against COVID-19 Mpro was demonstrated through a progress curve [95]. The curve's shape provided insights into the mechanism of two-step irreversible inhibition. To understand the inhibitory mechanism of N3, Jin *et al*. conducted a crystal structure analysis of the COVID-19 Mpro complexed with the compound at a resolution of 2.1 Å [96, 97]. Similar to N3, α-ketoamides have also been identified as potent inhibitors of the Mpro of both α- and β-coronaviruses, as well as the 3C proteases of enteroviruses [97].

### 4.1 Structure-based Design of Drugs for Target CoV Main Proteases

Nearby quarter of hundred coronaviruses (CoVs), which are significant pathogens instigating widespread illnesses, are found in the genus coronavirus. By linking 4 crystal buildings and homologous replicas representing each of the 3 genomic collections of the species Coronavirus, which was revealed that the prime CoV proteases (Mpro or 3CLpro) are mandatory for viral inheritable factor replication and expression and share a decidedly well-maintained substratum recognition compact [98, 99]. Too, all coronaviruses have a greater degree of maintenance at the active positions of Mpro, S1, S2, and S4. By inspecting the substrate-linking compact of SARS-CoV Mpro, a stoppage that goal SARS-CoV-2 Mpro can be designed and created (PDB ID 2H2Z). Dai *et al*. cast off an aldehyde as a new-fangled weapon to generate a covalent linkage through cysteine while creating a new inhibitor. To increase the activity, they also added cyclohexyl or 3-fluorophenyl to P2, and they added indole groups to P3 to create new H ties with S4, which enhances the drug-like capabilities [100]. A sedentary SARS-CoV Mpro (C145A) modification's crystal structure served as the source for manufacturing the cyclic peptide stoppage known as UCI-1. The C-terminal autolytic dividing position of the SARS-CoV Mpro, an intrinsically occurring Mpro substratum, is mimicked in the project of UCI-1. In UCI-1, an AEPA ([4-(2-amino-ethyl) phenyl]-acetic acid) group is used to connect the carboxyl-end of the P2' rest to the amino-end of the P2 rest, resulting in a cyclophane. AEPA's (2-amino-ethyl) phenyl set is intended to fill the S3' pocket and substitute for a phenylalanine adjacent part at position P3'. Additionally, studies indicate that UCI-1 looks harmless to humanoid embryonic kidney corpora at quantities that obstruct Mpro, but then again, it exhibits reduced activity compared to other Mpro inhibitors. Structure-based designs of drugs (SBDD) software are increasingly being cast off due to the target's structural elucidation [101, 102]. The fundamental phases of SBDD are delineated for understanding in Fig. (**1**). Diverse complementary computer-generated transmission and docking approaches can be used to find additional potential 3CLpro inhibitors from the ZINC15 library that are not yet certified active substances. Then, utilizing SBDD [103], these compounds might be further improved. However, one study improved the substratum to engender a cyclical peptide restraint inside the Mpro dynamic section by using the UCSF Chimera program [104, 105] after starting with the X-ray crystal construction of SARS-CoV Mpro [104]. With AutoDock Vina [106], this prototypical was, in conclusion, assessed by docking the restraint to Mpro of SARS-CoV-2.

### 4.2 SBDD for Target PLpro

Because PLpro also can get rid of ubiquitin and interferon-encouraged DNA segment 15 (ISG15) from host-organism proteins, it helps coronaviruses evade host innate immune responses [107]. GRL-0617 inhibits SARSCoV-2 PLpro in three ways: by curtailing viral reproduction in infected cells, maintaining the antiviral interferon pathway, and hindering the virus's cytopathogenic action [108]. Latent SARS-CoV PLpro restraint with 3,4-dihydro-2H-pyran fractions and naphthalene coupled by an NHCO joiner was recently developed. Another study was the first to do thorough commotion reporting of SARS-CoV-2 PLpro consuming HyCoSuL, an innovative chemical technique, and it identified the molecular principles governing PLpro substrate selectivity [109]. After that, powerful restraints (VIR251 and VIR250) that showed great fussiness against SARS-CoV-1 PLpro and interrelated SARS-CoV-2 PLpro were developed and biochemically described in comparison to other proteases. Furthermore, it was unexpectedly found that the P4 amino acids of VIR251 and VIR250 reside on mutual sides of the expansive S4 compact of SARS-CoV-2 PLpro, which will encourage the advancement of innovative medications. HyCoSul is grounded on the suggestion that SARS-CoV-2-PLpro is identical to SARS-CoVPLpro, although there are not abundant statistics available regarding SARS-CoV-2-PLpro. Abundant investigations have established the apo form, as well as composites with ADP-ribose (ADPr), 2-(N-morpholino) ethane sulfonic acid (MES), and AMP, of the SARS-CoV-2 ADP-ribose phosphatase realm (ADRP). Based on structure-based investigations, researchers have presented a reliable method to find prospective small-fragment restraints with apo crystals diffusing to microscopic resolution [110].

### 4.3 SBDD for Target Spike Glycoprotein

As early as 2003, a coronavirus was determined to cause SARS [111]. The most recent SARS-CoV-2 in 2019 has been found to have a striking resemblance to SARS in terms of both structure and function [112, 113]. The categorization resemblance between the COVID-19 genetic data from isolates from India, Italy, China, Nepal, and the USA and the humanoid SARS-CoV German isolate is about 60%, whereas with bat SARS-CoV it is 79-80% [114]. In contemplation for SARS-CoV-2 to tie to host receptors and enter cells and cause COVID-19 infection, spike glycoprotein (S) is essential [115, 116]. To investigate medicines against the receptor-binding dominion (RBD) of SARS-CoV-2, a virtual screening strategy, *in-silico* pharmacophore modeling, in-pathway, and technique have been applied. First, the pharmacophore is designed using Ligand Scout, the traditionalist range is cast off as a prototype, and the 3D structure of the RBD is raised. Then, prime composites are marked off by the Cambridge, ZINC, Drug Bank, and TIMBLE records. The selected prime composites are then docked molecularly and the interaction residues are visualized using AutoDock Vina. All the lead chemicals that were produced are just preliminary findings; further clinical and investigative testing is still needed. Angiotensin-converting enzyme 2 (ACE2), a cell sheath receptor, is now known to be crucial for the access of SARS-CoV-2 inside the cells [117]. The RBD arbitrates the interaction of ACE2 with SARS-CoV-2 S. To achieve this, the RBD, SARS-CoV-2, and a structure-based ACE2 variant dataset were integrated, yielding an overall 242 structural representation. These representations can be employed to develop new drugs and to train people about how ACE2 perceives the SARS-CoV-2 S protein [118]. The adjacent chains of K537 and E619 are known to form two important salt bridges. COVID-19 can be efficiently treated with medications created to stop the creation of these salt bridges, but because the protein complex is so straightforward, it is believed to also apply to the trimeric S protein [119]. Table **1** provides an overview of SBDD research against the outbreak of COVID-19 for many aimed proteins of SARS-COV-2. Amino acid and peptide transporters can also move tiny peptides across the cell membrane [120]. To block ACE, numerous short peptide compounds have been created [121, 122]. Selecting and combining the structures of decidedly dynamic molecules to create innovative peptide analogs is another potential technique [123, 124]. For dealing with COVID-19, Table **1** lists potential structurally connected medication designs.

### 4.4 SBDR Approaches

Different computational-based approaches have been proposed for managing various diseases, including COVID-19 [125]. In the specific context of COVID-19 management, several DR methods have been developed and can be categorized based on their computational techniques.

#### 4.4.1 ML-based Methods

Machine learning (ML) approaches are commonly employed to predict drug-target interactions (DTIs) such as drug-protein, drug-pathway, and drug-gene interactions. These models utilize experimentally validated DTIs to predict the likelihood of a significant interaction between a drug and a specific target. In the case of SARS-CoV-2, ML models can be applied to identify potential medications that can inhibit key targets of the virus, including the S protein, Mpro, and RdRp (RNA-dependent RNA polymerase) [126, 127]. ML techniques have been widely utilized in screening drugs for COVID-19, demonstrating their ability to provide accurate predictions compared to other technical methods [128]. However, it is worth noting that the effectiveness of these techniques is highly reliant on the availability of substantial and diverse datasets. Consequently, it is essential to validate the predicted results using secondary approaches such as *in vivo*, *in vitro*, and/or *in silico* experiments. This validation process ensures the reliability and credibility of the ML predictions in the context of drug screening for COVID-19 [129].

#### 4.4.2 NB Methods

Network-based approaches have emerged as promising techniques for managing diseases by understanding the connections between different biological elements, such as genes, proteins, and microRNAs [130]. In the context of combating COVID-19, researchers have focused on elucidating the protein-protein interactions (PPIs) between SARS-CoV-2 proteins and host cell proteins [131]. Subsequently, graph theory algorithms have been employed to analyze the resulting network. In one study, key nodes within the network were identified, and potential inhibitor drugs were explored as means to target these key nodes and potentially disrupt the viral infection process [132]. These network-based approaches have demonstrated significant potential in comprehending the intricate interactions between biological elements and identifying potential therapeutic targets for various diseases, including COVID-19 [132]. Complementary exposure component patterns were employed in a study [133] to discover two drugs, referred to as A and B, that exhibit the potential for enhanced effectiveness when used in combination rather than as individual therapies for treating a specific disease. However, it should be noted that the use of these approaches may not be suitable during the early stages of an emerging disease like COVID-19, as many aspects of such diseases remain unknown [134].

#### 4.4.3 Hybrid Methods

To achieve precise and practical outcomes, some studies have employed a combination of the aforementioned methods, leveraging their respective advantages. For example, in one study, researchers generated a model using machine learning (ML) techniques to predict drug-protein interactions. This model was then utilized to identify potential interactions between existing medications and SARS-CoV-2 proteins. To validate the predicted outcomes, a molecular docking analysis was subsequently conducted. This integrated approach aimed to uncover probable interactions between drugs and SARS-CoV-2 proteins. In another study, researchers sought to discover potential anti-SARS-CoV-2 medicines by employing a variety of bioinformatics tools. These tools incorporated the concepts of protein-protein interaction (PPI) networks and molecular docking. By combining these methods, the study aimed to identify promising compounds that could potentially exhibit antiviral activity against SARS-CoV-2 [135].

#### 4.4.4 Structural Proteins-related Studies

In recent research, significant attention has been directed toward studying the non-structural proteins of SARS-CoV-2. However, there have also been investigations utilizing SBDR (structure-based drug design) approaches to inhibit the structural proteins of the virus, aiming to understand their roles in preventing COVID-19. In one such study, the E (envelope), M (membrane), and N (nucleocapsid) proteins of SARS-CoV-2 were specifically targeted. The researchers conducted docking experiments to assess the impact of 548 natural and synthetic compounds known for their antiviral properties. To obtain the necessary data, the protein sequences and 3D structure of the N protein were obtained from the GenBank and PDB databases, respectively. Chemical information regarding the compounds was retrieved from the PubChem and SelleckChem databases. A web tool called I-TASSER was utilized to generate 3D models of the M and E proteins, as their structural information was not available. Subsequently, several commonly used software suites, including YASARA, CASTp, Molsoft, and Autodock Vina, were employed for energy minimization, prediction of active site residues, determination of pharmacokinetic traits, and calculation of binding affinity scores, respectively. The findings suggested that specific combinations such as doxycycline and rutin, caffeic and ferulic acids, and simeprevir and grazoprevir might exhibit inhibitory effects on the E, M, and N proteins, respectively [136].

## 5 SERIOUS VALUATION OF EARLY CLINICAL, EXPERIMENTAL, AND COMPUTATIONAL STUDIES ON DRUG REPURPOSING COUNTER TO COVID-19

**Fig. (2):**
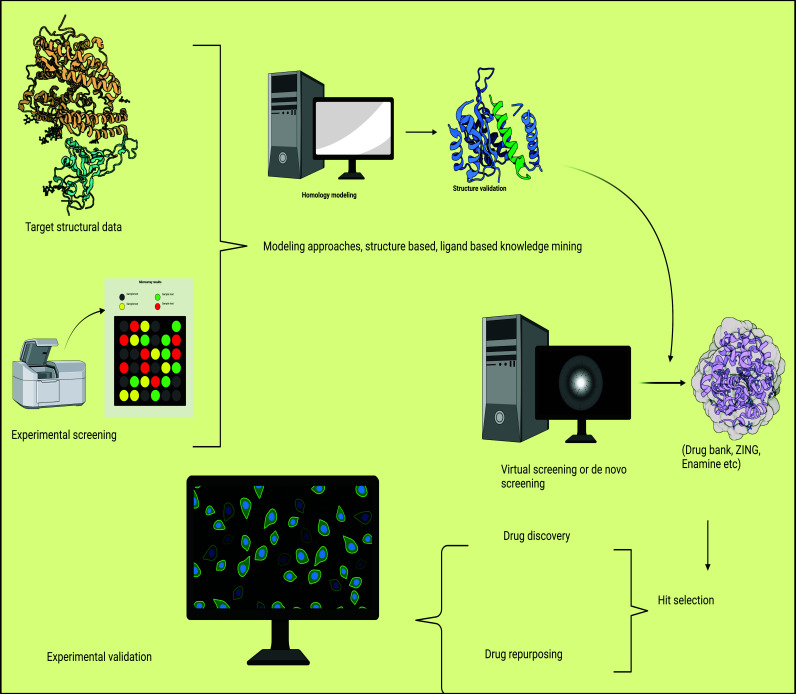
Summary of crucial progresses in CADD for COVID-19.

Scientists from all around the world felt a sense of urgency as a result of COVID-19's appearance. Numerous researchers with varied informative and qualified educations have transmitted their computational or investigational work to identify COVID-19 therapeutic contenders. Several articles reported substances with modest micromolar *in-vitro* action against SARS-CoV-2 in the premature phases of the outburst. Most of this research only evaluated a small number of unique chemical entities and mostly used FDA-approved medications. Grander canopies were later conducted, and various triumphs were screened using infected animal or humanoid cells. To present, the accomplishment of hundreds of anatomically unalike small particles has been surveyed in virus-infected cells. As many of these researchers have supplied information to support the construction of computational models and hit validation, we will briefly cover a few below [112, 136]. A durable context for open discipline, plus facts discussion, data allocation, and the practice of open-source software, is provided by the turf of computer-aided designing of the drug (CADD), which personifies alliance between experimental, computational and clinical professors as well as merging of multidisciplinary, goal-oriented styles towards unearthing and advancement of innovative and compelling medicines. The trained usage of techniques and faithfulness to CADD's superlative practices accelerate experimental success and permit the quick creation of effective, experimentally verified therapeutic candidates (Fig. **2**).

## 6 KNOWLEDGE PULLING OUT TOOLS FOR COVID-19 DRUG DISCOVERY

The brutality of coronavirus outbursts spurred academics, universities, publishers, companies, and regulators to embrace uncluttered knowledge and FIAR (Findable, Interpretable, Accessible, Reusable) info initiatives [137] to understand the illness better and discover a treatment as soon as possible. Several planned and amorphous COVID-19 facts foundations have been presented to the community to expand the usage of data drawing out procedures besides Artificial Intelligence (AI) - fast-tracked gears for COVID-19 drug finding [138]. Some noteworthy illustrations are covered in the subsequent segment.

## 7 THE PRACTICE OF KNOWLEDGE GRAPH STYLES FOR COVID-19 DRUG REPURPOSING

Biomedical information displays the intention to make available an advanced interpretation of the interactions sandwiched between illness entities (warning signs, ontologies, *etc*.), biotic goals (inheritable factors, protein complexes, proteins, nucleic acids), and biochemical things (experimental and trial drugs, device mixes, *etc*.) (KG) [139]. These affairs can be in a straight line to get from controlled data resources like medical and biomedical records or amorphous data resources like a mass of systematic documents and patents using text mining techniques with the help of machine learning techniques. Entity recognition NLP methods can be used to create KGs from unstructured data. This determines which objects in the text share common primary articles; relation taking out, which locates germane subject-predicate-object triplets in writing; and relation standing, which rates the accuracy of the facts hauled out either manually or automatically) [140]. Once such a KG has been created, specialists can investigate the correlations to identify crucial new medication targets or substances connected to a disease process. To forecast new connections between objects in the graph, supplementary ML approaches, such as tensor factoring, diagram convolutional neuronic networks, and logical extrapolation algorithms, can be laboring for graph comprehensiveness. One of the furthermost striking illustrations of a KG was fashioned by Benevolent AI for drug repurposing counter to COVID-19. This KG contained within a sizable store of controlled medical facts, plentiful links gleaned from systematic works using a variety of ML algorithms, and more [141]. A bespoke diagram was finished, and a sub-diagram about SARS-CoV-2 was isolated from empowering expert evaluation to locate a medication effective against COVID-19 [142]. This study shows that the virus attaches to host cells through the ACE2 receptor, which is grounded on the superiority of lung AT2 alveolar epithelial cells. ACE2 simplices clathrin-intermediated endocytosis, which is encouraged by the NAK family of enzymes consisting of GAK and AAK1. It was discovered that the rheumatoid arthritis medication baricitinib is a NAK restraint with high plenty of plasma absorption to choke AAK1. Therefore, it was presented for clinical investigation. Baricitinib, a JAK-STAT signaling restraint, was also anticipated to be convenient in dropping the high cytokine levels (cytokine squall) seen in the COVID-19 patient role. Based on the KG, it was too anticipated to have an untroublesome adjacent consequence profile and a short chance of drug interactions.

## 8 KNOWLEDGE-BASED DISCOVERY OF SYNERGETIC DRUG BLENDS FOR COVID-19

**Fig. (3):**
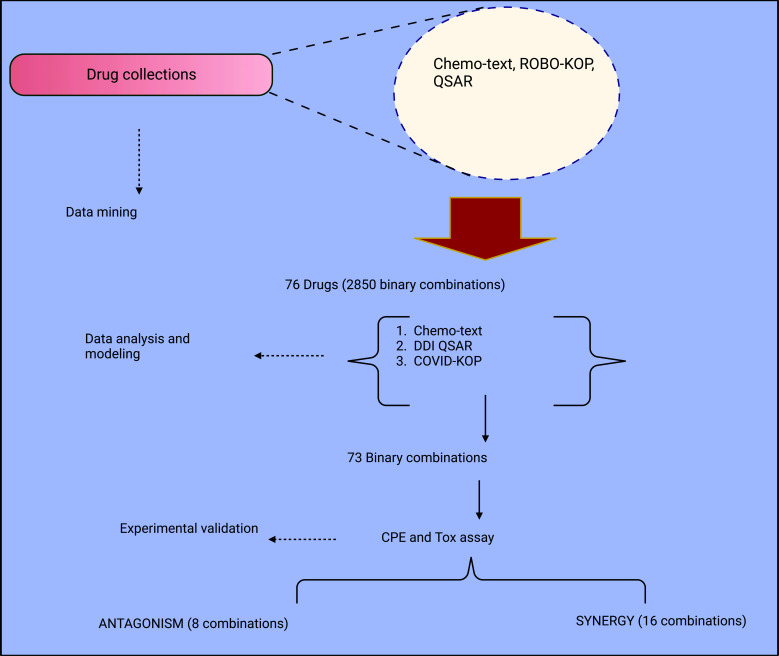
Study design for recognizing drug blends.

The synergistic interaction of medications creates a wealth of beneficial treatment prospects. The anti-HIV medication combinations' outstanding efficacy and the synergism of many other DAA drugs emphasize how crucial it is to investigate amalgamation remedies for COVID-19. For this reason, cutting-edge AI knowledge can be appointed as operative analytics gears to investigate pharmacological blends that work in concert to treat SARS-CoV2 [143, 144]. A thorough explanation of this study design can be found in Fig. (**3**), where the initial footstep involves the usage of a grouping of text pulling out (using Chemotext), knowledge pulling out (using ROBOKOP/COVID-KOP facts tables), [145] and machine learning (QSAR) [146] gears to find current medications that may have anti-SARS-CoV-2 [147] activities. 76 dissimilar medicine contenders were documented as portions of likely groupings based on the preliminary findings. These medications have 2850 possible unique component combinations; to maximize the likelihood that these combinations work synergistically, it was prioritized to match medications with various modes of action or that target various stages of the viral life cycle [148]. Thus, 95 ternary combinations of 15 medicines and 281 two-fold groupings of 38 preparations were selected for further analysis. Next, it was determined whether specific chemicals had been studied in combination before and whether adverse drug-drug interactions might be forecasted using the *in-silico* conduit integrating Chemotext, just established COVID-KOP, [149], and QSAR mock-ups of important drug-drug communications [150]. The final prioritized list had 32 medications and 73 preferred two-fold mixtures for SARS-CoV-2 *in vitro* testing [151] (Fig. **3**).

## 9 THE IMPORTANCE OF ACCURACY IN EXPERIMENTAL AND COMPUTATIONAL APPROACHES TO COVID-19 DRUG FINDING

As we have repeatedly said in this learning, the key steps in the modeling road map—statistics curation, archetypal building, computer-generated transmission, and tentative investigation of computational winners—must be carried out accurately and according to the best standards. The scientific literature has extensively discussed model development and data curation best practices. Different methods for curating chemical [152] and biological [153] data have been covered elsewhere. Similarly, numerous renowned publications and reports [154, 155] have explored optimal practices for computational model validation. Highly referenced reviews [156, 157] also noted the significance of objectivity in statistics curation and archetypal authentication. Still, we alleged it was critical to high spot here the unsurpassed practices that should be trailed when submitting and testing drugs that stand up from *in-silico* studies as high-confidence unsure winners, particularly with repute to molecular dockage.

## 10 COVID-19 AND OPEN SCIENCE

A team of doyens who have fervent they are skilled breathes to molecular demonstrating, and drug unearthing was responsible for the conception and development of this contribution. The in-depth analyses of CADD and QSAR unsurpassed practices have been laboriously crafted [158]. Here, we gave a general overview of the CADD strategies that have been employed to contest SARS-CoV-2, one of the most exciting destructive sicknesses acknowledged to mankind. For COVID-19, we go over reputable and recently developed computational procedures in addition to conversing the unsurpassed practices for statistics dealing out and algorithm finishing. We also emphasized the significance of open science and teamwork. The COVID-19 problem has significantly boosted scientific collaboration and openness because transparency speeds up research. All revelries can back up new-fangled facts extra hurriedly and effectively by sharing information and ideas with few constraints, which reduces the need for duplication. Three projects sum up our readiness and attempt to forgo conventional scientific limitations (such as the requirement to get a patent, safe as houses examine capital, or raise our academic profile): Uncluttered information, open source, and open entree [159].

## 11 THE IMPACT OF EPIDEMICS ON CARBON EMISSIONS

The COVID-19 pandemic and the subsequent implementation of lockdown measures in many countries had a notable impact on carbon emissions, although the relationship between the two is complex. The temporary decline in greenhouse gas emissions can be attributed to the restrictions imposed on travel, industrial activities, and the overall economic slowdown. These measures resulted in a significant reduction in the burning of fossil fuels and, consequently, a global decrease of approximately 6% in CO2 emissions in 2020, as estimated by the International Energy Agency (IEA) [160]. During the COVID-19 pandemic, there was an initial decrease in carbon emissions, which had some positive environmental effects. One of the notable impacts was the improvement in air quality, particularly in heavily polluted areas. The reduction in economic activities and travel restrictions led to a temporary decrease in water pollution and noise pollution as well. Nevertheless, as restrictions were eased and economic activities resumed, carbon emissions rebounded, offsetting these short-term improvements. The pandemic did, however, initiate discussions and actions regarding long-term structural changes in various sectors, such as transportation and remote work, aiming to address environmental concerns and create a more sustainable future. The COVID-19 pandemic has had a notable impact on the energy sector, offering an opportunity to embrace more sustainable practices and reduce carbon emissions in the long run, as argued by several experts. The pandemic has affected the energy sector in different ways. On one hand, there has been a decline in the demand for fossil fuels due to reduced travel and industrial activities. On the other hand, the renewable energy sector has displayed resilience and continued growth, even in the face of the pandemic [161]. The decline in oil prices and the subsequent economic downturn have presented significant challenges for renewable energy investments and hindered the progress towards adopting clean energy in certain regions. As governments devise strategies for economic recovery, they face a crucial decision regarding the balance between short-term economic stimulation and long-term sustainability. Numerous experts strongly advocate for prioritizing green recovery plans that emphasize investments in renewable energy, energy efficiency, and low-carbon infrastructure. Such measures are seen as vital in ensuring a resilient and sustainable future. Climate change and carbon emissions remain ongoing challenges, despite the current focus on the COVID-19 pandemic. The effects of climate change can have indirect consequences for public health, including an increased frequency and severity of extreme weather events, water and food insecurity, and the potential spread of infectious diseases. It is crucial to prioritize addressing climate change alongside managing the COVID-19 crisis to ensure global health and build resilience. While the pandemic temporarily reduced carbon emissions due to lockdowns and reduced economic activity, it is important to recognize that such measures are not sustainable long-term solutions to the underlying issue of climate change. Efforts and policies aimed at transitioning to a low-carbon economy and mitigating the impacts of climate change require a long-term commitment. While research at the biophysical level is crucial for studying the link between climate and infectious diseases, it is equally important to recognize the role of socioeconomic factors in both climate change and the spread of such diseases. Currently, many countries are prioritizing the reduction of greenhouse gas emissions and are implementing strategies such as “total control and trading” to achieve this goal. However, it is important to approach these issues holistically, considering the complex interplay between climate change, socioeconomic factors, and the spread of infectious diseases [162].

## 12 DISCUSSION

Computational approaches can aid drug development in several ways and accelerate the process. While typical traditional formulation development takes ~10 years, computational techniques could speed up this process and reduce costs. Granting swapping to an unalike medication is an alternative mutual process of treating SARS-CoV-2. We posit that truly impactful computational tools must deliver actionable, experimentally testable hypotheses enabling the discovery of novel drugs and drug combinations and that open science and rapid sharing of research results are critical to accelerating the development of novel, much-needed therapeutics for COVID-19. COVID-19 is recurrently not pointedly restrained by prior remedies; thus, creating medications to combat SARS-CoV-2 is more rational and desirable and could serve as a durable foremost line of defense for forthcoming coronavirus-related remedies. Technology improvements in current ages have extended structural biology and made it possible to determine more protein structures, speeding up the process of medication design. A new drug's design is unstated to cost the outmoded pharmaceutical commercial US$2600 million, with a 90% disaster rate sandwiched between clinical trials and sanctions [163]. The advancement of AI-based and structure-based medication projects has significantly slashed time and expense while increasing creativity. Identifying target-protein and target-drug interactions is also thoughtful for AI-based and structure-based drug design. While AI-based constructions habit machine knowledge, bottomless knowledge procedures, and computer-generated transmission to pinpoint the central fragment, SBDD depends more on the three-dimensional structure to improve medicinal molecules with broader chemical space. CADD techniques have been employed to identify potential drug targets in the SARS-CoV-2 virus, the virus responsible for COVID-19. By studying the viral proteins and their interactions with human cells, researchers have been able to identify specific viral proteins as potential targets for therapeutic intervention. CADD techniques, such as virtual screening and molecular docking, enable researchers to rapidly screen large databases of compounds and predict their interactions with viral proteins. This speeds up the process of identifying potential drug candidates, allowing scientists to focus on the most promising ones. Overall, CADD has significantly contributed to the development of potential drugs and therapeutic strategies for COVID-19. By combining computational approaches with experimental validation, researchers can expedite the drug discovery process and potentially identify effective treatments to combat the pandemic. Despite its computational nature, CADD still requires significant computational power and resources to carry out extensive virtual screening, molecular docking, and simulations. The time and resource constraints can limit the scale and scope of CADD studies, potentially affecting the comprehensiveness of the drug discovery process. While CADD plays a vital role in accelerating drug discovery, these limitations highlight the need for a multidisciplinary approach that combines computational methods with experimental validation and a deep understanding of the underlying biology of COVID-19.

## CONCLUSION

The current results suggest that CADD approaches relying on ligand- and structure-based methodologies could be used with success to predict inhibitory action against SARS-CoV-2. The COVID-19 outbreak and the pandemic situation have hastened the research community to design a novel drug and vaccine against its causative organism, the SARS-CoV-2. The spike glycoprotein present on the surface of this pathogenic organism plays an immense role in viral entry and antigenicity. This accelerated computer research- aided drug designing, especially in the field of structure-based drug designing. This review summarizes various structure-based drug design approaches applied to this SARS-CoV-2 spike protein and its findings. Specifically, it is focused on different structure-based approaches such as molecular docking, high-throughput virtual screening, molecular dynamics simulation, drug repurposing, and target-based pharmacophore modeling and screening. CADD methods have been instrumental in identifying existing drugs that could potentially be repurposed to treat COVID-19. By screening libraries of approved drugs or compounds in clinical development against viral targets, researchers have identified several candidates that could be repurposed to target SARS-CoV-2. It's important to note that while CADD has accelerated the drug discovery process, experimental validation, and clinical trials are necessary to confirm the efficacy and safety of potential drug candidates. CADD serves as a valuable tool in guiding and prioritizing the drug discovery process, but it does not replace the need for rigorous experimental testing.

## Figures and Tables

**Fig. (1):**
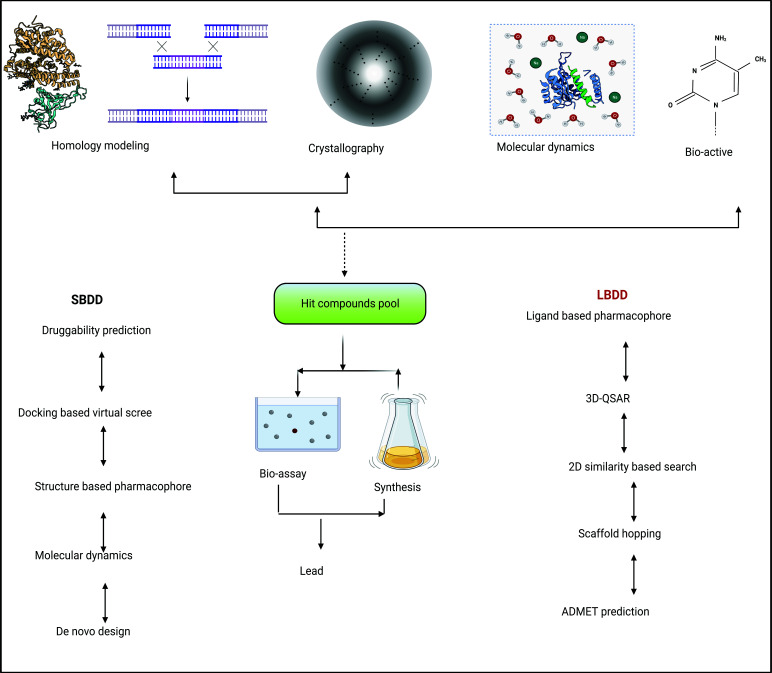
Old workflow of structure-based drug design (SBDD) along with ligand-based drug design (LBDD).

**Table 1:** Structure-based approaches for designing of drug.

**Aimed Protein**	**Explanation**	**Merits**	**Pharmacophore or Imaginable ** **Designing Drugs**	**Refs**
Spike of glycoprotein	The well-preserved section was cast-off as a template to project a pharmacophore by Ligand Scout.	Interacting rests were envisioned.	6-(1H-imidazol-1-yl)-N-[1-(3,4,5-trifluoro phenyl) ethyl] pyrimidin-4-amine.	[115]
Spike of glycoprotein	242 structural representations of modifications of human ACE2 bound to the receptor binding dominion (RBD) of the S protein was manufactured and polished their boundaries with HADDOCK.	The impacts of these discrepancies on the 3D structure of the protein and its composite with RBD have been scientifically studied.	https://kastritislab.github.io/human-ace2-variants/.	[116]
Spike of glycoprotein	Mutations of E619D and K537Q compacted their adjacent chain lengths and eradicated this duo of salt bridges.	The screened fragment is skilled in hindering the construction of the main pair of salt bridges.	The adjacent chains of K537 and E619.	[117]
Mpro	The aldehyde sets of 11a and 11b are covalently bonded to cysteine 145 of Mpro.	Mutually compounds exhibited good pharmacokinetic assets *in vivo*, between which 11a has lesser toxicity.	11a in addition to 11b.	[118]
Mpro	The cyclic peptide restraint was premeditated to copy the conformation of a substratum at a C-end autolytic cleavage position of Mpro.	The restraint is active against Mpro *in vitro* and is nontoxic on the way to human cells in media.	A first-in-class cyclical peptide restraint.	[119]
Mpro	3CL proteases have a chief part in polyprotein processing during replication.	The period up to sanction as a therapeutic counter to SARS-CoV-2 could expectantly be summarized.	Hydrogen-bond donors or acceptors of compact amino acids.	[120]
PLpro	Probable SARS-CoV PLpro restraint encompassing 3,4-dihydro-2H-pyran moieties and naphthalene allied *via* -NHCO- joiner have been considered.	The premeditated ligands counter to the receptor SARS CoV-2 Papain-like protease (PLpro) have solid binding attraction and inhibition latent.	Naphthalene grounded SARS-CoV PLpro restraint.	[121]
PLpro	Viral papain-similar cysteine protease (PLpro, NSP3) denotes a hopeful target for the advancement of antiviral drugs.	SARS-CoV-2-PLpro harbours deISGylating actions analogous to SARS-CoV-1-PLpro, but its capability to hydrolyse K48-linked Ub chains is moderated.	VIR250 as well as VIR251.	[122]
PLpro	Macrodomains may support to cut the viral load and enable regaining.	A vigorous system has been settled to categorize latent small-molecule restraint for structure-based experimentations.	The anomeric C atom.	[123]

## LIST OF ABBREVIATIONS

CADD: Computer-Aided Designing Drug
CNB: China National Biotech
DR: Drug Repurposing
DTIs: Drug-Target Interactions
ML: Machine Learning
NO2: Nitrogen Dioxide
PPI: Protein-Protein Interaction
RBD: Receptor Binding Dominion
SBDD: Structure-Based Designs Drugs
SBDR: Structure-Based DR
